# Nucleostemin: New Stabilizer of ARF

**DOI:** 10.18632/oncoscience.282

**Published:** 2015-12-30

**Authors:** Bo Cao, Hua Lu

**Affiliations:** Department of Biochemistry and Molecular Biology, Tulane Cancer Center, Tulane University School of Medicine, New Orleans, Louisiana, 70112, USA

**Keywords:** ARF, nucleostemin, ULF, ubiquitination, nucleophosmin

As an essential nucleolar protein for ribosomal assembly and protein production, nucleolar GTPase nucleostemin (NS) is often highly expressed in actively proliferative cells, including stem cells and cancer cells, and therefore thought to play an oncogenic role in various types of human cancers [1]. However, given the heterogeneity of cancer cells, imbalanced expression of NS could trigger distinct events to regulate cell proliferation in different genetic backgrounds. For instance, in wild type p53-harboring osteosarcoma cell line U2OS, increased expression of NS inhibits MDM2 E3 ligase activity toward p53 and thus activates p53, resulting in G1 cell cycle arrest, while knocking down NS also activates p53 indirectly by causing ribosomal stress that induces the interaction of ribosomal protein L5 and L11 with MDM2 and consequently inhibits MDM2 activity toward p53 [2, 3].

**Figure 1:**
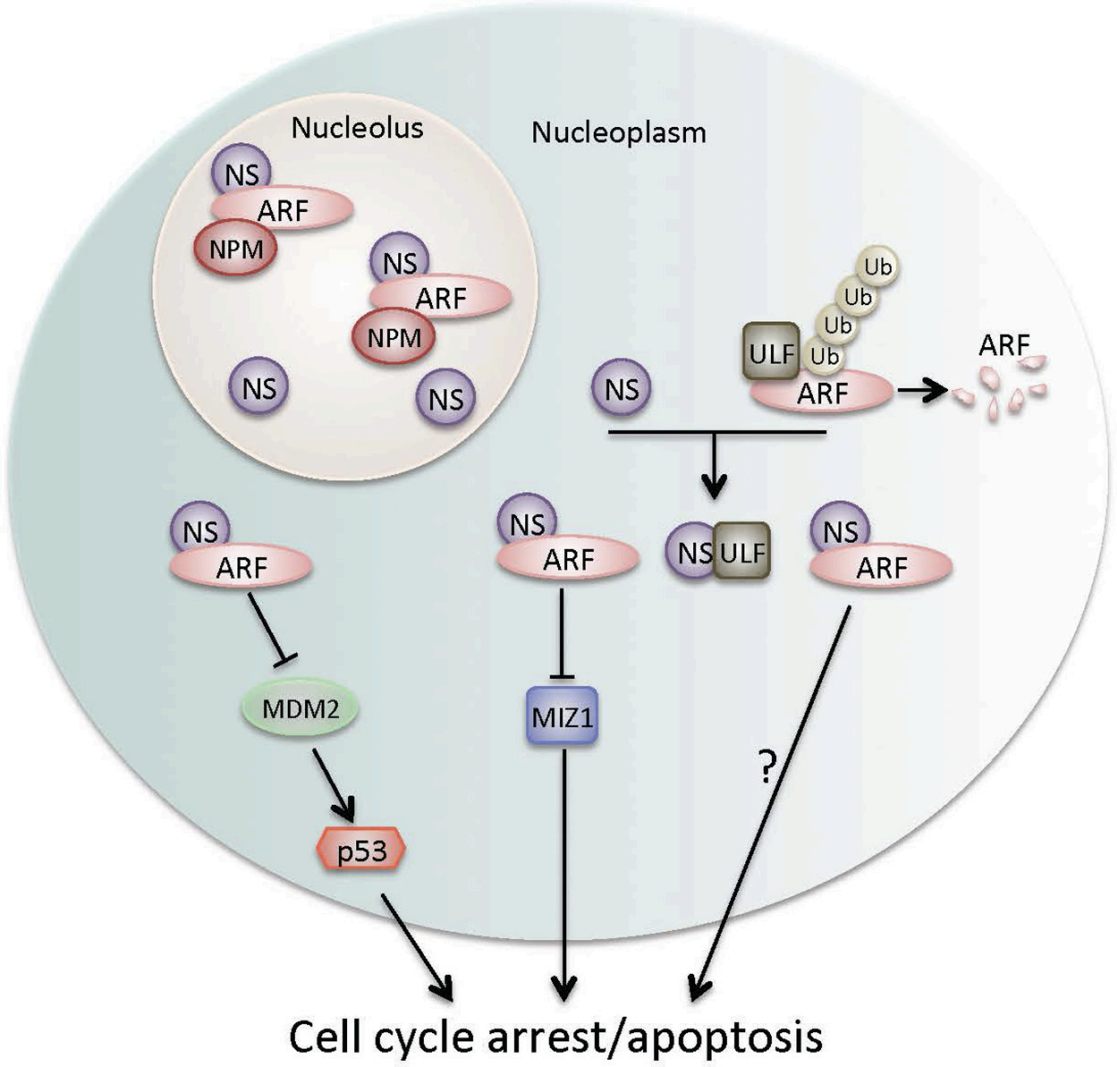
Nucleostemin regulation of pathways involved in cell cycle arrest and apoptosis

Making this NS-engaged regulation more complicated is our recent identification of the alternative reading frame (ARF), an upstream p53 activator in response to oncogenic stress [4], as another NS-binding protein through affinity purification coupled with mass spectrometry [5]. This binding occurs at the N-termini of both NS (amino acid 1–268) and ARF (amino acid 1–65) [5]. Interestingly, although these sites are also required for binding to nucleophosmin (NPM), which was previously shown to prevent ARF from proteosomal degradation by sequestering ARF in the nucleolus [6], NPM and NS do not appear to compete with each other for ARF binding [5]. Instead, NPM and NS are highly likely to form a stable complex with ARF in the nucleolus, working together to protect ARF [5]. However, our data further revealed that NS is not required for NPM to keep ARF in the nucleolus, but responsible for stabilization of nucleoplasmic ARF dissociated from the ARF-NPM complex resulting from depletion of NPM [5]. These findings indicate that abnormal expression of NS, in addition to causing oncogenic effects under certain circumstances and inducing p53 as an counteraction, could also stabilize tumor-suppressor ARF by enhancing the binding of NPM to ARF in the nucleolus and/or by directly interacting with ARF in the nucleoplasm when NPM is absent, providing an alternative surveillance to prevent aberrantly expressed NS-mediated tumor cell proliferation and transformation (Fig. 1).

More interestingly, similar to NPM [6], NS is able to bind to ULF and inhibit its E3 ligase activity toward ARF [5], as ULF was identified as an E3 ligase responsible for ARF polyubiquitination and proteosomal degradation in the nucleoplasm, which was inhibited by NPM [6]. Different from NPM, the NS inhibition of ULF appears to occur in the nucleoplasm [5], as NS reduces the interaction between ARF and ULF, inhibiting ULF-mediated ARF polyubiqutination and degradation (Fig. 1). Consequently, enforced expression of NS in p53/MDM2 double knockout, but not in p53/ MDM2/ARF triple knockout, MEF cells induces G1 cell cycle arrest. Therefore, in addition to activation of p53 by ARF in response to ULF suppression as previously reported [6], our observations suggest that ARF accumulation by NS overexpression through disruption of the ULF-ARF interaction has growth inhibitory effects independently of p53 [5]. As ULF is primarily located in the nucleoplasm [6], one remaining question is how different cellular signals that mediate NS expression levels or NS shuttling between the nucleolus and the nucleoplasm contribute to NS inhibition of ULF. A previous study demonstrated that depletion of guanine nucleotides or GTP not only regulates the cellular distribution of NS, but also mediates NS degradation [7]. Would the altered levels of cellular GTP contribute to the regulation of the NS-ARF-ULF pathway?

ULF has been proposed as a molecular sensor to oncogenic stresses by maintaining ARF at a low level in unstressed cells, while allowing transcription-independent induction of ARF when oncogenes, such as c-Myc, are activated, leading to activation of p53 [6]. Our study demonstrates similar induction of ARF by NS through interaction with ULF, but a distinct mechanism by which the growth inhibitory effect of activated ARF is p53- independent. As the role of ARF as a tumor suppressor has been attributed majorly to MDM2 inhibition and subsequently p53 activation, further in-depth investigation is necessary to discover other downstream events responsible for the growth inhibition effect of ARF independently of p53 (Fig. 1). MIZ1 transcription factor is another protein associated with ARF. ARF binds to MIZ1 and inactivates its function by facilitating the assembly of a complex containing MIZ1/MYC/trimethylated H3K9, resulting in repression of genes favorable for cell survival [8]. Would NS regulate this MIZ1-ARF complex formation and its cellular and functional consequences (Fig. 1)? Additional clinic-relevant questions are whether the expression of NS is correlated with ARF expression in tumor samples, and whether this correlation is significant in cancer progression or prognosis?
